# Supplementary material for: A marine sponge-derived lectin reveals hidden pathway for thrombopoietin receptor activation
Source: Nat Commun. 2022 Nov 25;13:7262. doi: 10.1038/s41467-022-34921-2 (PMC9700728; doi:10.1038/s41467-022-34921-2)
Supplement: Supplementary file 1 — Supplementary Info File #1 [file 41467_2022_34921_MOESM1_ESM.pdf]

Supplementary Information

A marine sponge-derived lectin reveals hidden pathway for thrombopoietin receptor activation

Hiromi Watari[1]†, Hiromu Kageyama[2]†, Nami Masubuchi[3], Hiroya Nakajima[1], Kako Onodera[2], Pamela J. Focia[4], Takumi Oshiro[5], Takashi Matsui[5,6], Yoshio Kodera[5,6], Tomohisa Ogawa[2], Takeshi Yokoyama[2], Makoto Hirayama[7], Kanji Hori[7], Douglas M. Freymann[4], Misa Imai[3], Norio Komatsu[3,8,9], Marito Araki[3]*, Yoshikazu Tanaka[2]*, Ryuichi Sakai[1]*

*Corresponding authors. Email: Ryuichi Sakai <ryu.sakai@fish.hokudai.ac.jp>; Marito Araki <m-araki@juntendo.ac.jp>; Yoshikazu TANAKA <yoshikazu.tanaka@tohoku.ac.jp>
†These authors contributed equally to this work.

[1]*Graduate School of Fisheries Sciences, Hokkaido University; Hakodate, Japan.*
[2]*Graduate School of Life Sciences, Tohoku University; Sendai, Japan.*
[3]*Laboratory for the development of therapies against MPN, Juntendo University Graduate School of Medicine; Tokyo, Japan.*
[4]*Department of Biochemistry & Molecular Genetics, Feinberg School of Medicine, Northwestern University; Chicago, USA.*
[5]*Department of Physics, School of Science, Kitasato University; Sagamihara, Japan.*
[6]*Center for Disease Proteomics, School of Science, Kitasato University; Sagamihara, Japan.*
[7]*Graduate School of Integrated Sciences for Life, Hiroshima University; Higashi-Hiroshima, Japan*
[8]*Department of Advanced Hematology, Juntendo University Graduate School of Medicine; Tokyo, Japan.*
[9]*Department of Hematology, Juntendo University Graduate School of Medicine; Tokyo, Japan.*

Supplementary Tables

Supplementary Table 1. Affinities and thermodynamic parameters of binding [a]

protein	ligand	5mM $CaCl_2$	K_D (μM)[b]	ΔH (kcal mol^{-1})[c]	ΔS (cal mol^{-1} K^{-1})[c]	ΔG (kcal mol^{-1})[c]	K_A (M^{-1} x 10^4)[c]	K_D in the previous report (μM)
rThC	fucose	+	4.72 ± 0.25	-8.14	-2.94	-7.26	21.2 ± 1.12	
		-	NB	NB	NB		NB	
	mannose	+	66.2 ± 15.3	-0.76	16.6	-5.71	1.51 ± 0.35	
		-	NB	NB	NB		NB	
Q25K	fucose	+	NB	NB	NB		NB	
G132	fucose	+	NB	NB	NB		NB	
BC2LC-CTD	fucose	+	NB	NB	NB		NB	
	mannose	+	17.7 ± 0.32	-10.0	-11.9	-6.45	5.65 ± 0.42	37.4[(1)]
PAIIL	fucose	+	2.78 ± 0.30	-6.46	3.75	-7.58	35.9 ± 3.90	2.9 ± 0.03[(2)]
	mannose	+	78.7 ± 23.6	-9.46	-12.9	-5.62	1.27 ± 0.38	(Me-α-Man: 71 ± 3)[(2)]

Not bound is indicated as "NB".

a These parameters were determined in 20 mM HEPES-NaOH (pH 8.0), 200 mM NaCl, at 25 °C

b $K_D = 1/K_A$

c $-RT \ln K_A = \Delta H - T\Delta S = \Delta G$

Supplementary Table 2. Data collection and refinement statistics

		Native ThC	SeMet Substituted rThC	rThC in complex with Ca^{2+} and fucose	rThC in complex with Ca^{2+} and mannose	rThC Q25K in complex with Ca^{2+}
PDB ID		7F9F	7F91	7F9G	7FBL	7F9J
Beamline		APS BL 21-ID-F	Photon Factory BL-1A	Photon Factory NE3A	Photon Factory NE3A	Photon Factory NE3A
Data collection						
Space group		$P2_1$	$P2_12_12_1$	$P2_12_12_1$	$P2_12_12_1$	$P2_12_12_1$
Cell dimensions						
	a, b, c (Å)	42.46, 92.44, 58.54	41.80, 45.00, 109.91	41.52, 44.72, 108.58	41.57, 44.98, 109.26	41.66, 44.88, 109.56
	α, β, γ (°)	90, 102.8, 90	90, 90, 90	90, 90, 90	90, 90, 90	90, 90, 90
Resolution (Å) [a]		30.47 - 1.41 (1.43 - 1.41)	41.64 - 1.40 (1.49 - 1.40)	41.35 -1.33 (1.41 - 1.33)	41.59 - 1.42 (1.50 - 1.42)	38.94 - 1.10 (1.17 - 1.10)
R_{sym} (%) [a]		9.9 (64.2)	9.6 (66.3)	5.7 (37.7)	7.7 (50.3)	5.3 (65.8)
$<I/\sigma(I)>$ [a]		25.15 (2.17)	11.28 (2.14)	26.05 (5.64)	22.27 (4.64)	21.83 (2.70)
Completeness (%) [a]		99.9 (99.7)	99.9 (99.6)	99.8 (98.7)	99.7 (98.7)	99.4 (96.6)
Redundancy [a]		5.7 (4.4)	7.04 (7.07)	9.60 (9.48)	9.51 (9.31)	9.19 (7.57)
Wilson B-factor (Å^2)		10.3	13.6	9.1	9.6	10.9
Refinement						
No. of reflections used in refinement		81704	41580	47088	39532	83639
No. of reflections used for R_{free}		1950	2080	1642	1369	2900
R_{work} / R_{free}		0.169 / 0.194	0.175 / 0.200	0.155 / 0.168	0.162 / 0.182	0.185 / 0.197
No. atoms						
	Protein	3854	1940	2009	2017	2023
	Ligand	2	0	26	27	2
	Water	799	247	323	317	332
R.m.s deviations						
	Bond lengths (Å)	0.005	0.005	0.005	0.007	0.005
	Bond angles (°)	0.89	0.89	0.93	0.97	0.93
Average B-factor (Å^2)		15.4	17.0	11.6	11.1	14.3
	Protein	13.5	16.1	10.0	9.7	12.8
	ligand	7.9	-	14.7	7.4	18.1
	Water	24.9	24.3	21.6	20.4	23.2
Ramachandran plot						
	Favored (%)	97.67	97.67	97.67	97.28	98.05
	Allowed (%)	2.33	2.33	2.33	2.33	1.95
	Disallowed (%)	0	0	0	0.39	0

a The values in parentheses refer to data in the highest resolution shell.

Supplementary Table 3. Primers used for the construction of MPL and rThC derivatives

Primer Name	Primer Sequence
MPL N117Q Fw	5'-GTGTTCCTACAGCAGACTCGGACTCAGCGAGTCC-3'
MPL N117Q Rev	5'-CGAGTCTGCTGTAGGAACACATTCTTCACCCAG-3'
MPL N178Q Fw	5'-GATCCCAAGCAGTCCACTGGTCCCACGGTCATACAG-3'
MPL N178Q Rev	5'-CCAGTGGACTGCTTGGGATCTCTGGGGCCATAGC-3'
MPL N298Q Fw	5'-GACCTGAAGCAGGTTACCTGTCAATGGCAGCAAC-3'
MPL N298Q Rev	5'-CAGGTAACCTGCTTCAGGTCCAAGGTAAAGCATTGC-3'
MPL N358Q Fw	5'-CAAGTCACGACAGGACAGCATTATTCACATCCTTG-3'
MPL N358Q Rev	5'-GCTGTCCTGTCGTGACTTGAAGTGGCAGCGAGAG-3'
rThC Fw	5’-TCCGTGCAAAGCTCGCGCAATGAGAGC-3’
rThC Rev	5’-CGAGCTTTGCACGGACGCATTCAGGCT-3’
rThC G132 Fw	5’-TTCGCGGTGGCTAACTCGAGCACC-3’
rThC G132 Rev	5’-GCTCGAGTTAGCCACCGCGAACTAAC-3’

Supplementary Notes

Supplementary Note 1

Amino acid sequence determination.

We first identified a preliminary 131-amino acid sequence of native ThC (nThC) isolated from the sponge using mass spectrometry and Edman degradation after peptic digestion (Supplementary Figure 3a). A draft ThC based on this sequence assigned lysine (K) to the 25th amino acid residue. However, unlike nThC, the draft ThC failed to promote the proliferation of MPL activation-dependent Ba/F3-HuMpl, even at high concentrations (Supplementary Figure 3b). This lack of activity showed that at least one amino acid residue in the draft-ThC sequence was assigned incorrectly. Therefore, we performed X-ray crystal structure analysis of nThC and found that the 25th amino acid residue originally assigned as K in the draft-ThC sequence was glutamic acid (E) or glutamine (Q) (Supplementary Figure 3c). An LC-MS/MS experiment unambiguously identified this residue as Q (Supplementary Figures 4 - 6), and the complete amino acid sequence of ThC was determined.

Supplementary Note 2

A structure comparison between apo and sugar bound lectins.

In the ITC experiment, unfavorable entropic change was observed in rThC-fucose binding and PA-IIL-mannose binding, while PA-IIL-fucose binding was favorable in both enthalpy and entropy terms (Supplementary Table 1). In the reviewing process of this manuscript, one of the reviewers suggested some large conformational changes such as induced fit that might account for the difference in the thermodynamic differences. We thus tested this idea by comparing the crystal structures of apo-rThC, rThC-fucose, rThC-mannose, and apo- PA-IIL, PA-IIL-fucose, and PA-IIL-mannose. The RMSDs for the apo- and sugar-bound structures are very low in all cases (Supplementary Table 4), suggesting that there are no major global structural changes interpretable as induced fit which is typically taken into account when RMSD is greater than ~2 Å [1]. Some local structure changes, on the other hand, in both rThC and PA-IIL were observed, but only at residues around the sugar binding site upon fucose/mannose binding. However, no clear correlation between mannose and fucose and the structural change were found (Supplementary Table 4); that is, in the case of rThC, both fucose/mannose showed conformational changes compared with the apo form, whereas in the case of PA-IIL, mannose-bound forms showed only slight difference from the

apo-state, but the fucose-bound form appeared to have conformational change. However, no clear correlation between the conformational change and entropy term was found (Supplementary Table 4).

This result may suggest that it is difficult to interpret each interaction solely from the numerical thermodynamic properties obtained in each experiment. In fact, there is a report [2] that investigated in detail the correlation between sugar-binding activity and conformation of sugar-binding proteins (PA-IIL and CV-IIL) that are very similar to rThC. Sabin *et al* have also reported in binding between PA-IIL and fucose, slight gain in entropy was observed, while that with methyl-α-fucose, unfavorable entropy was evident. [3] In both cases, no obvious correlation between the structure and thermodynamic profiles were found and authors of both papers concluded that it is difficult to corroborate mechanistic basis at this point.

Supplementary Table 4. Conformational changes and entropy terms of lectins upon sugar binding.

		Fuc	Man
rThC	global[a]	0.21	0.17
	local[b]	large	large
	ΔS	-2.94	16.6
PA-IIL	global[a]	0.39	0.21
	local[b]	larger	slight
	ΔS	3.75	-12.9

a. RMSD (Å), rThC: apo vs fucose: 218 Cα atoms, apo vs mannose: 214 Cα atoms; PA-IIL (when comparing dimer):apo vs fucose: 215 Cα atoms; apo vs mannose: 200 Cα atoms

b. Judged from conformational difference between apo and sugar-bound state around the sugar binding sites in Supplementary Figure 1.

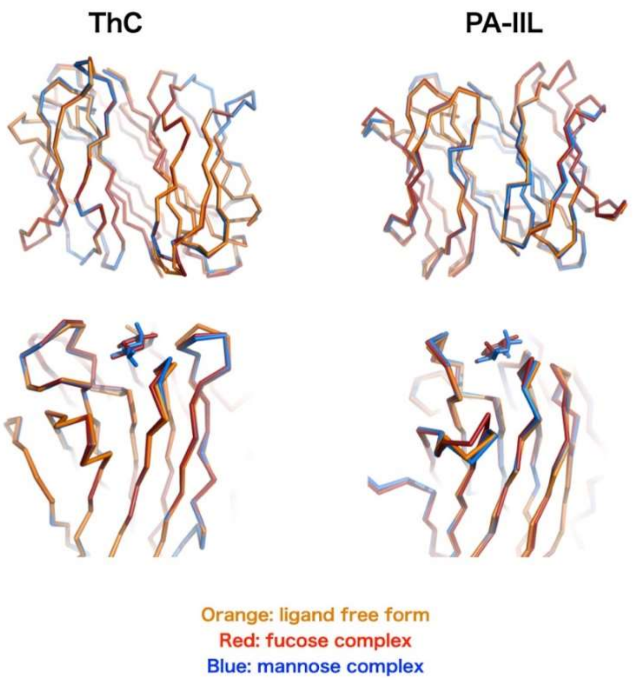

Supplementary Figure 1. Superimposition of rThC and PA-IIL ligand unbound (orange), fucose-bound (red), and mannose-bound (blue) states. The upper panel shows the global structure and the lower panel shows the local structure around the sugar binding site.

Supplementary Note 3

Crystal structure determination.

In the crystal structure of rThC–fucose, the electron density of fucose at one of the carbohydrate-binding sites was more obscure than that at the other (the average B-factor of all atoms in fucose was 8.4 and 22.6, respectively; Supplementary Figure 2). Moreover, mannose clearly bound to one sugar-binding site but not to the other. These results suggested that the binding of sugar to the two sites in the crystal milieu is not equivalent, likely due to neighboring effect in the process of crystal packing. However, the ITC thermogram showing a sigmoidal curve with a typical two-state transition indicated that the stoichiometry of ThC to sugar is 1:1 and two sugar-binding sites in the ThC dimer were occupied in solution. Therefore, the structure of the site with clear electron density was used in this manuscript for discussion of the interaction between sugar and ThC.

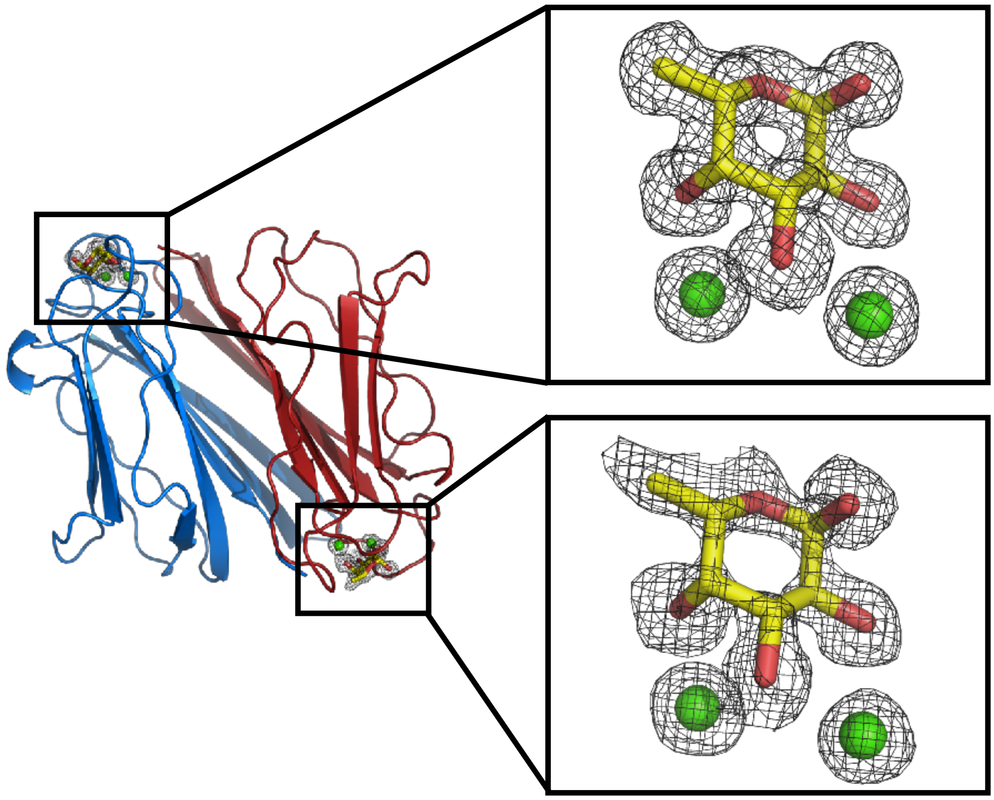

Supplementary Figure 2. Fo-Fc map (contoured at 1.3 σ) of the fucose (ball-and-stick model, yellow: carbon, red: oxygen) and Ca^{2+} ions (green ball) bound in the two sugar binding sites of rThC. In the case of ThC-fucose, the electron density at one of the carbohydrate binding sites was obscurer than the other (average of B-factor of all atoms in the fucose was 8.4 and 22.6, respectively).

Supplementary Figures

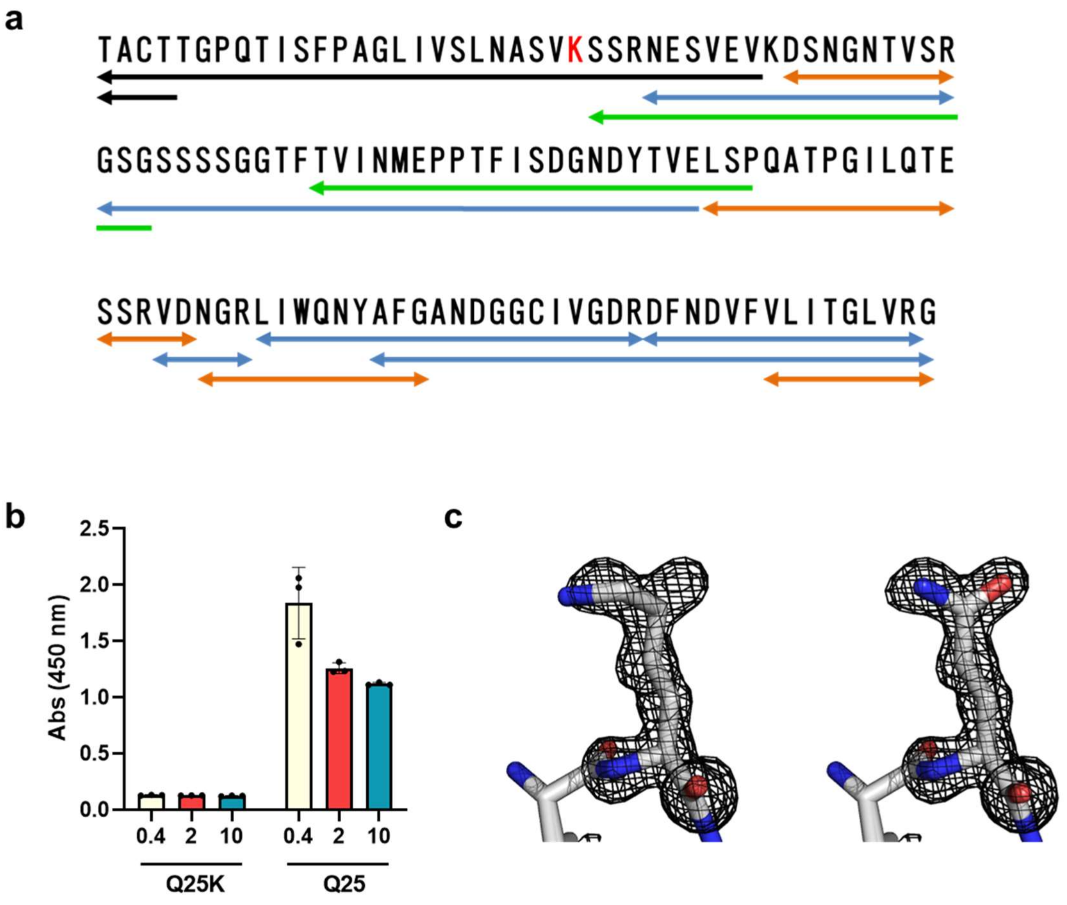

Supplementary Figure 3. Sequence determination of ThC

a Draft amino acid sequence of nThC. Amino acid sequence of native ThC was first determined on the basis of Edman degradation (black line), and MALDI-TOF MS/MS *de novo* sequecing data of chymotrypsin (green), trypsin, (blue), and V8 protease (orange) digests. The 25th residue was assignes to by lysine (red letter) for draft nThC (d-ThC). b Effect of rThC (Q25) and d-ThC (Q25K) on cell proliferation of Ba/F3-HuMpl cells. *n*=3 per data point. c Omit map of the 25th residue of nThC (contoured at 4.0 σ). Atomic models of Lys (left) and Gln (right) are in the omit map. Gln fits well (right) whereas Lys does not with the electron density (left).

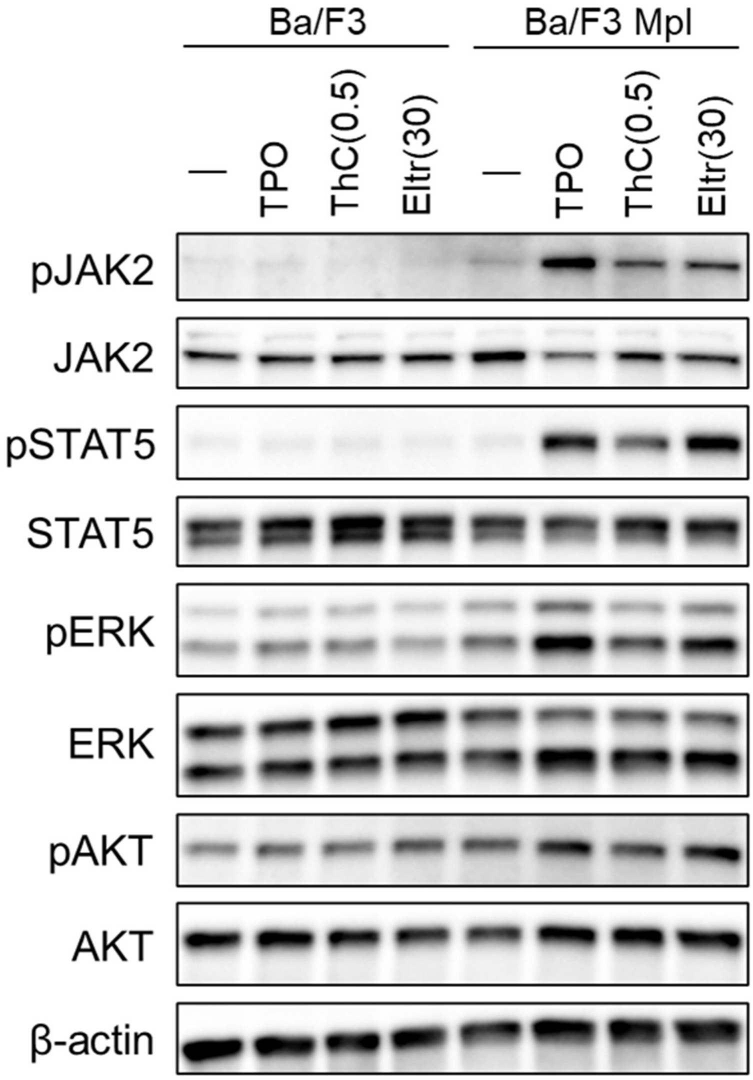

Supplementary Figure 4. Sequence determination of ThC continued
Immunoblot analysis at 24h after activation of Ba/F3 and Ba/F3-HuMpl cells upon steady-state activation by TPO and rThC. Three independent experiments were carried out.

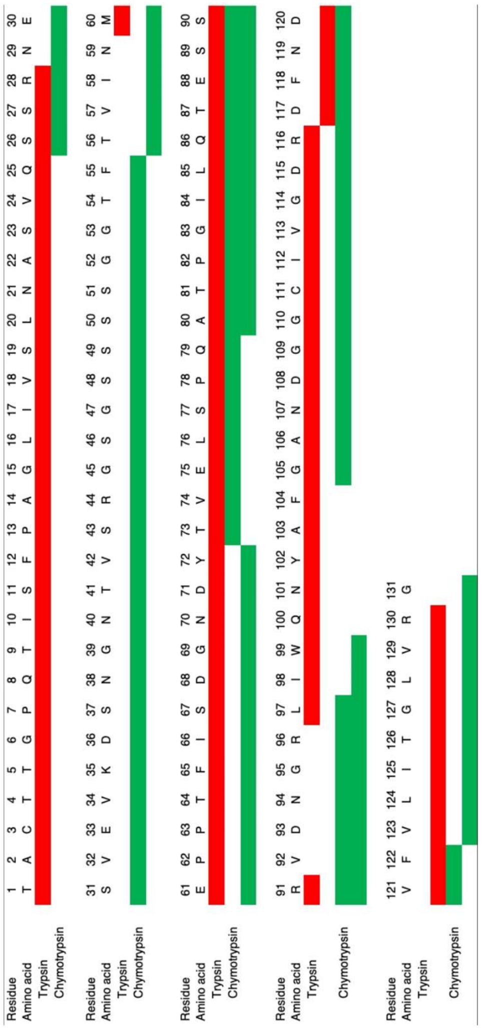

Supplementary Figure 5. Sequence determination of ThC continued
The amino acid sequence of nThC was re-examined by using ultrahigh resolution LC-MS/MS *de novo* sequencing. Peptide fragments, of which amino acid sequence were determined, generated by trypsin or chymotrypsin and shown red and green, respectively. Cys residue was treated with iodoacetamide to prevent from S-S bond reformation thus Cys residue was searched as Cys carbamidomethylation.

TAcTTGPQTISFPAGLIVSLNASVQSSR (res 1-28)

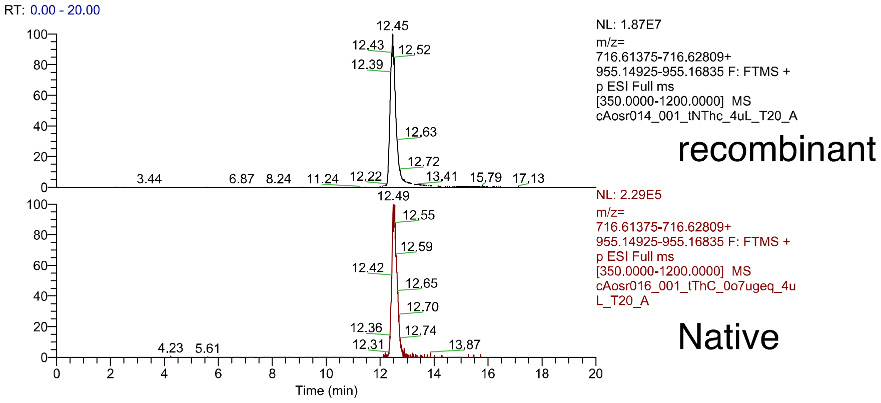

charge 4 (theoretical m/z=716.62092)
recombinant m/z=716.62140, RT=12.62 Δppm = 0.63 (PD)
Native

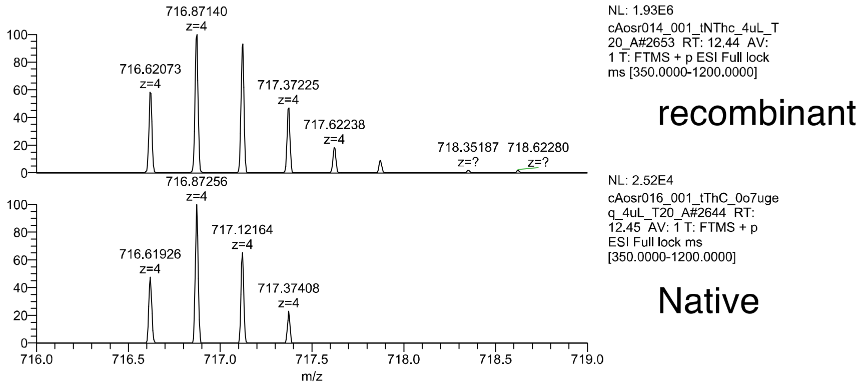

charge 3 (theoretical m/z=955.15880)
recombinant
Native m/z=955.15845, RT=12.38, Δppm=-0.41 (PD)

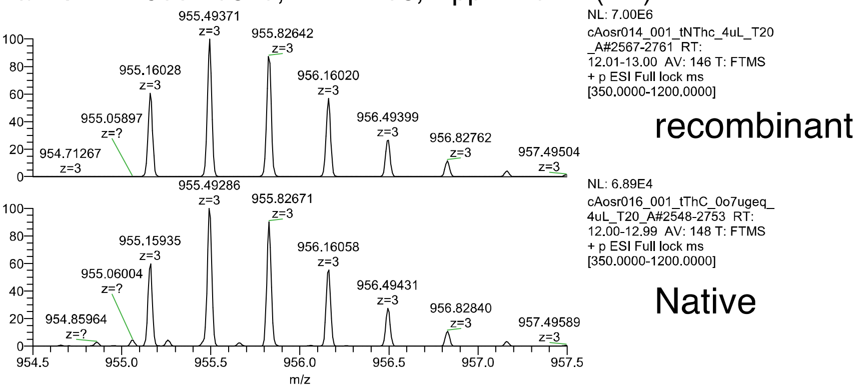

SSRNESVEVKDSNGNTVSRGSGSSSSGGTF (res 26-55)

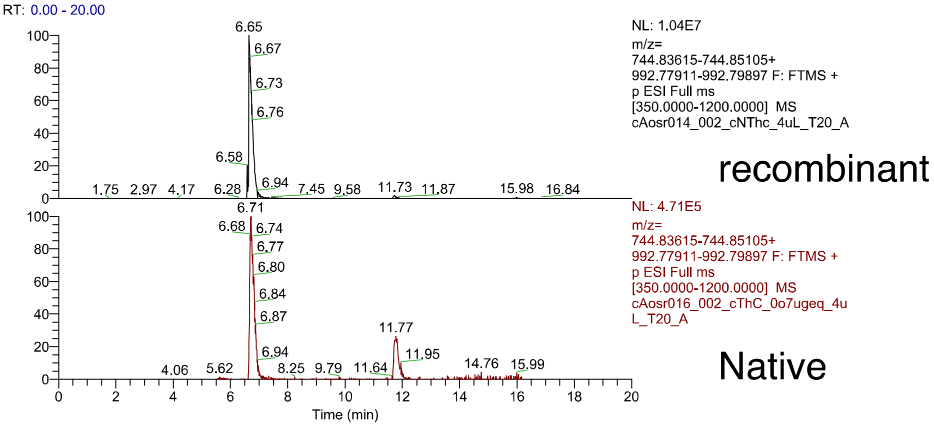

charge 3 (theoretical m/z=992.78904)
recombinant m/z=992.78967, RT=6.59, Δppm=0.60 (PD)
Native

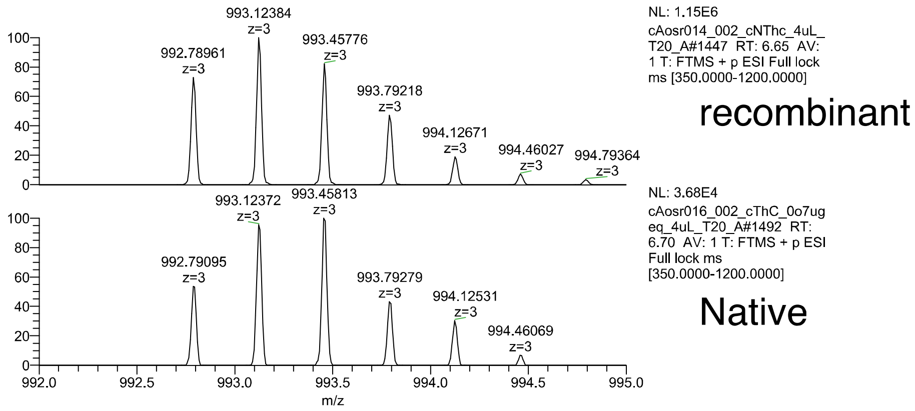

charge 4 (theoretical m/z=744.84360)
recombinant m/z=744.8442, RT=6.57, Δppm=0.8 (PEAKS)
Native m/z=744.8444, RT=6.71, Δppm=1.0 (PEAKS)

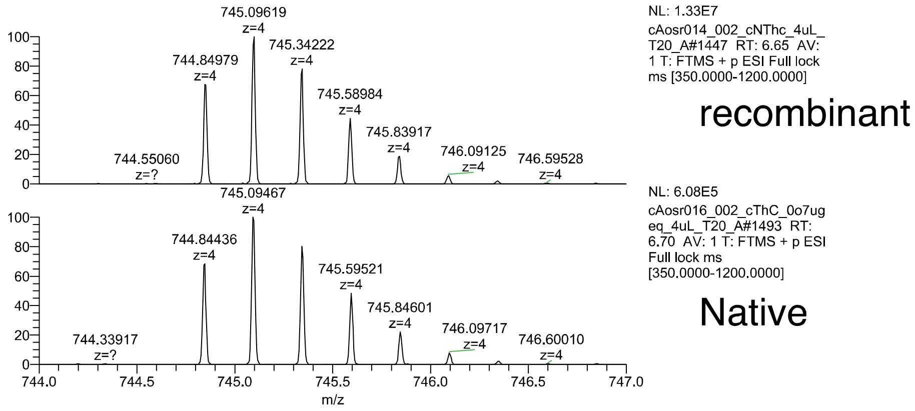

TVINMEPPTFISDGNDY (res 56-72)

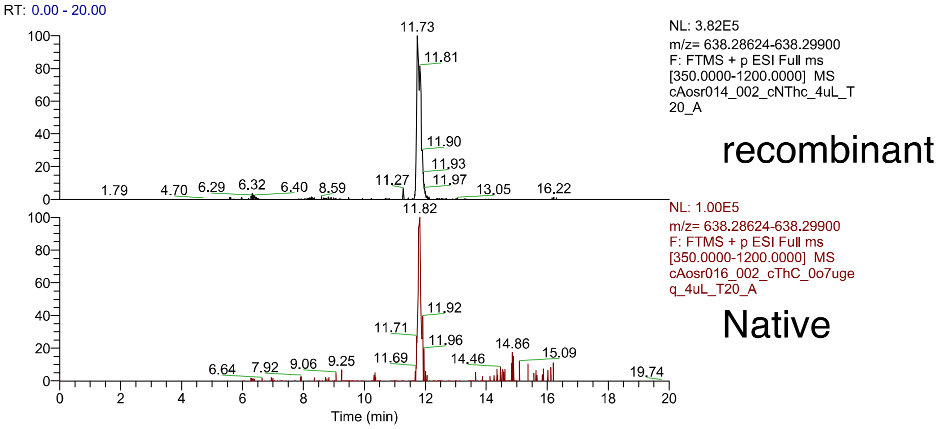

charge 3 (theoretical m/z=638.29262)
recombinant m/z=638.2927, RT=11.71, Δppm=0.2 (PEAKS)
Native m/z=638.2935, RT=11.80, Δppm=1.4 (PEAKS)

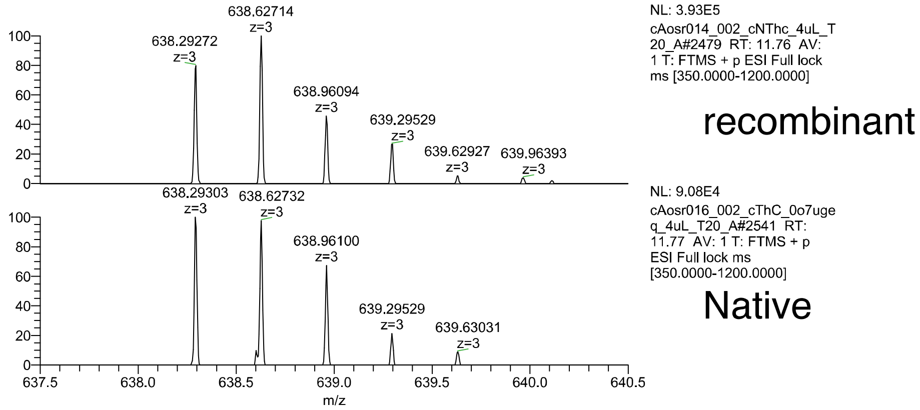

MEPPTFISDGNDYTVELSPQATPGILQTESSR (res 60-91)

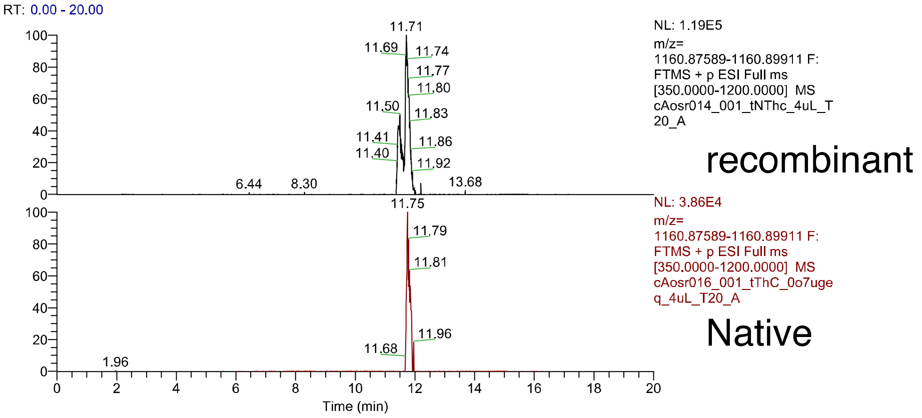

charge 3 (theoretical m/z=1160.88736)
recombinant
Native m/z=1160.8875, RT=11.75, Δppm=0.1 (PEAKS)

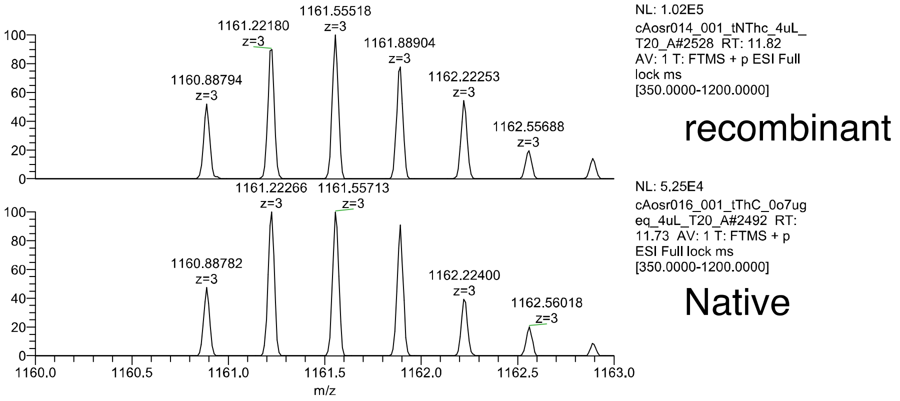

TVELSPQATPGILQTESSRVDNGRL (res 73-97)

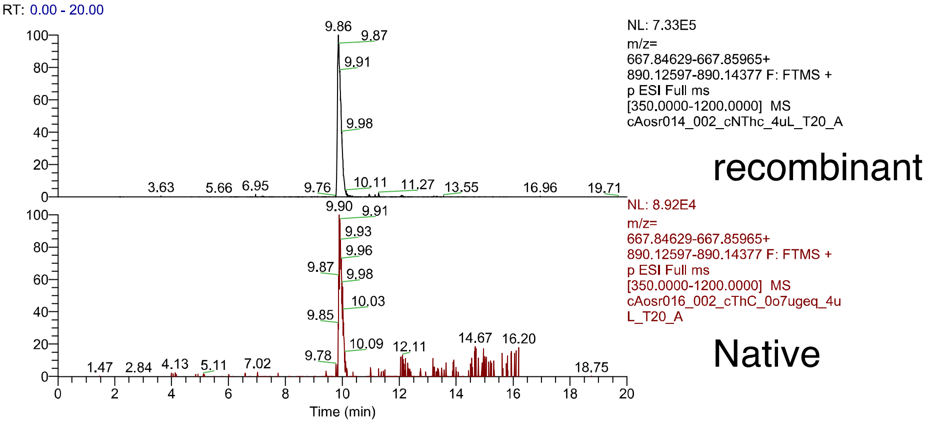

charge 4 (theoretical m/z=667.85297)

recombinant m/z=667.85278, RT=9.83, Δppm=-0.33 (PD)

Native

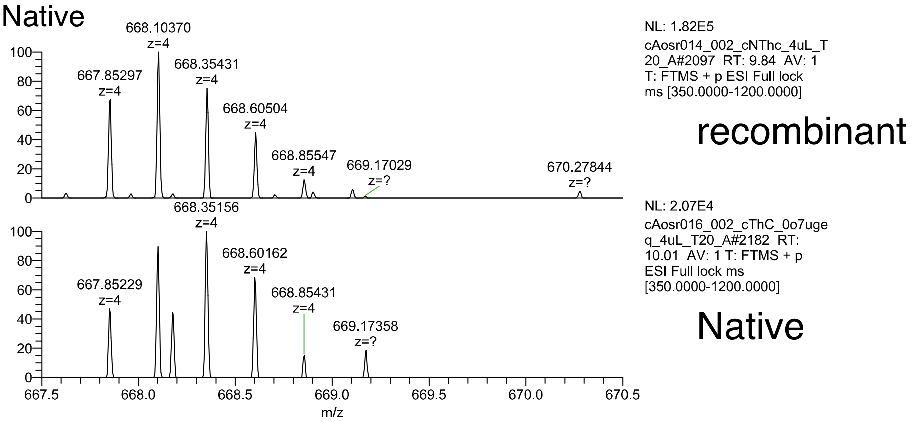

charge 3 (theoretical m/z=890.13487)

recombinant

Native m/z=890.13531, RT=9.89, Δppm=0.46 (PD)

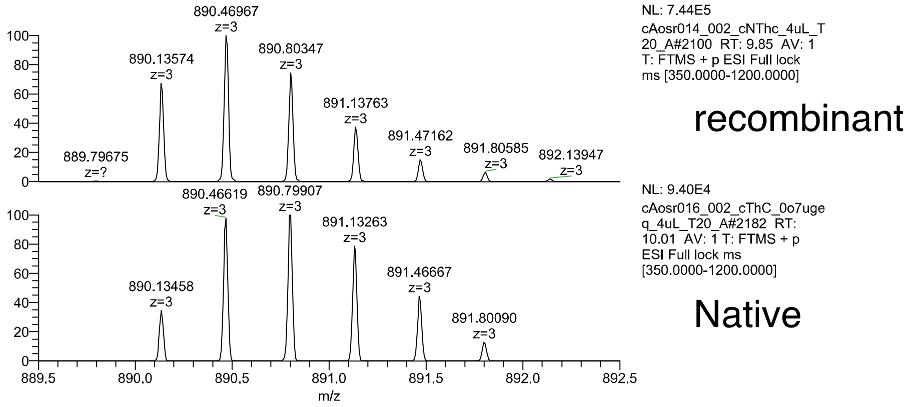

ATPGILQTESSRVDNGRLIW (res 80-99)

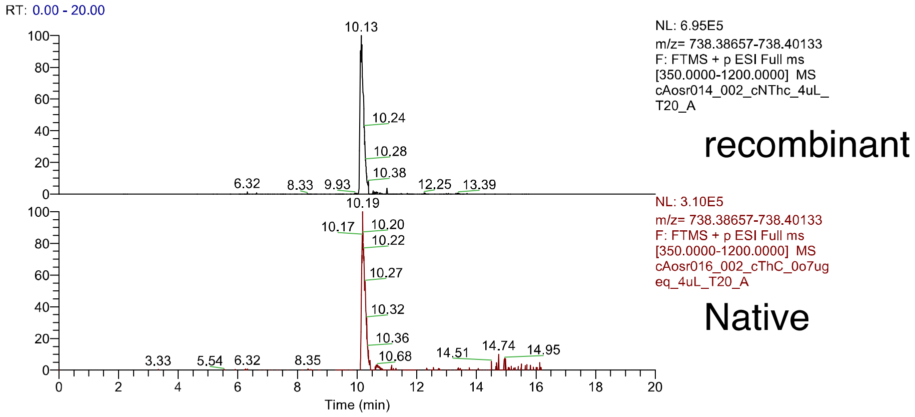

charge 3 (theoretical m/z=738.39395)
recombinant m/z=738.39441, RT=10.09, Δppm=0.57 (PD)
Native m/z=738.39429, RT=10.14, Δppm=0.41 (PD)

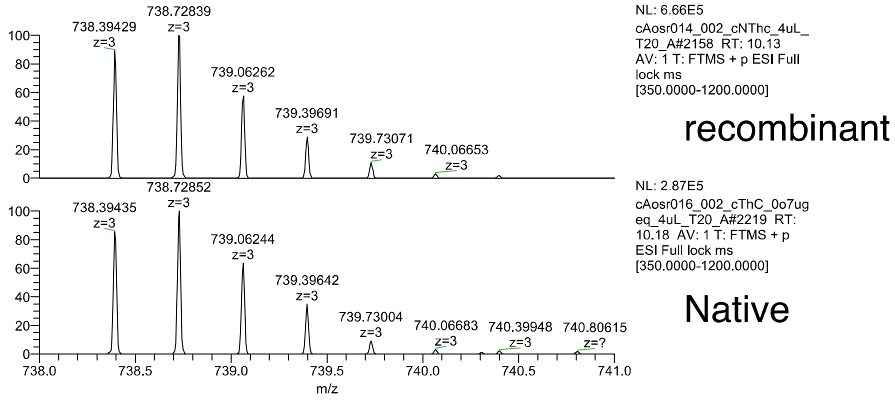

LIWQNYAFGANDGGcIVGDR (res 97-116)

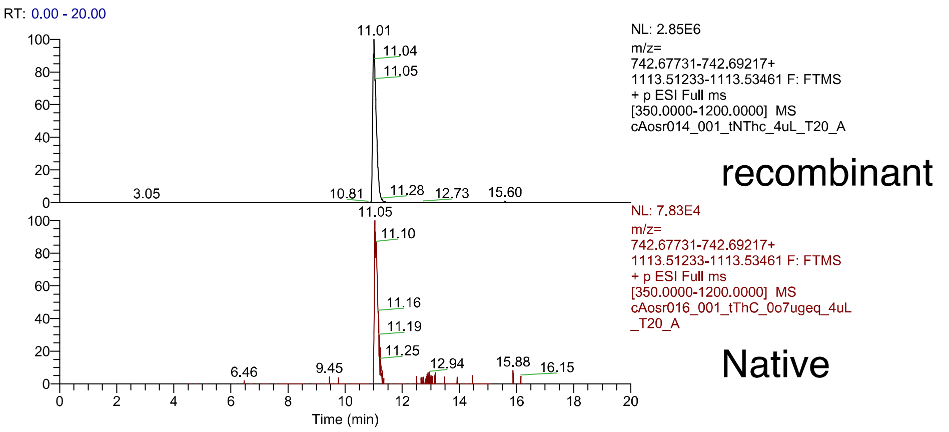

charge 3 (theoretical m/z=742.68474)
recombinant
Native m/z=742.68451, RT=11.06, Δppm=-0.36 (PD)

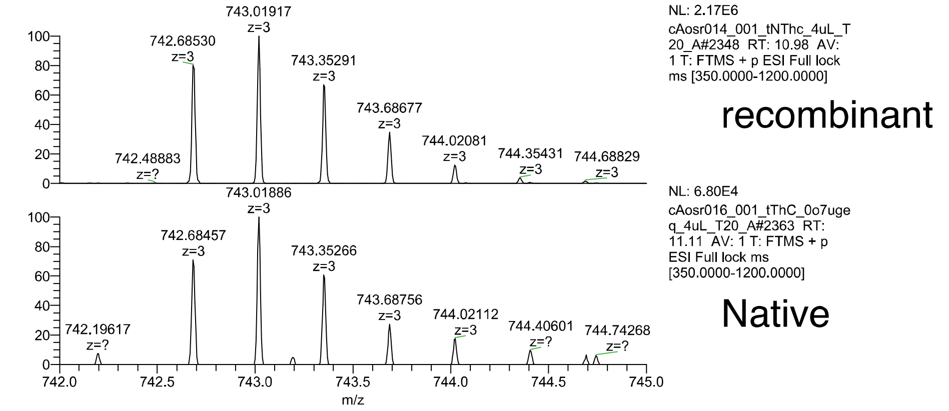

charge 2 (theoretical 1113.52347)
recombinant m/z=1113.52417, RT=10.96, Δppm=0.58 (PD)
Native

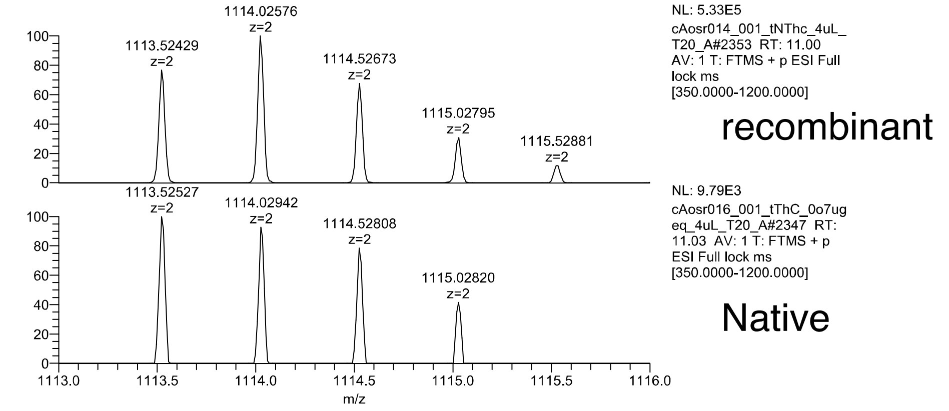

GANDGGcIVGDRDFNDVF (res 105-122)

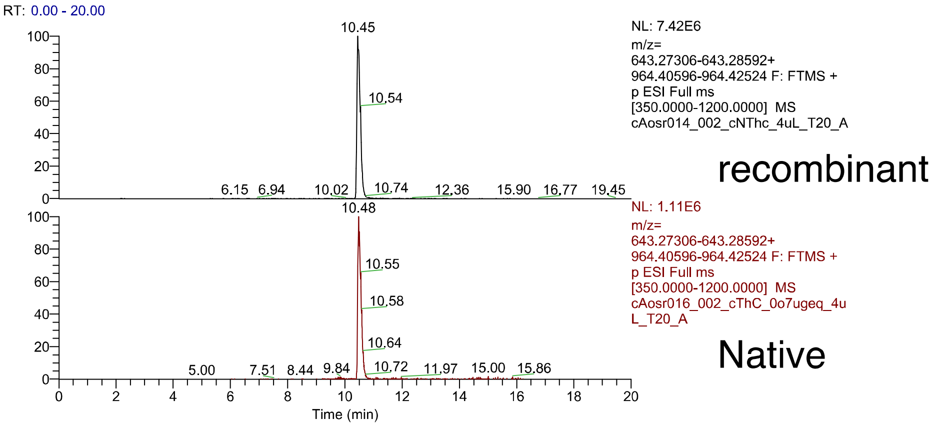

charge 2 (theoretical m/z=964.41560)
recombinant m/z=964.41559, RT=10.41, Δppm=-0.06 (PD)
Native m/z=964.41583, RT=10.43, Δppm=0.19 (PD)

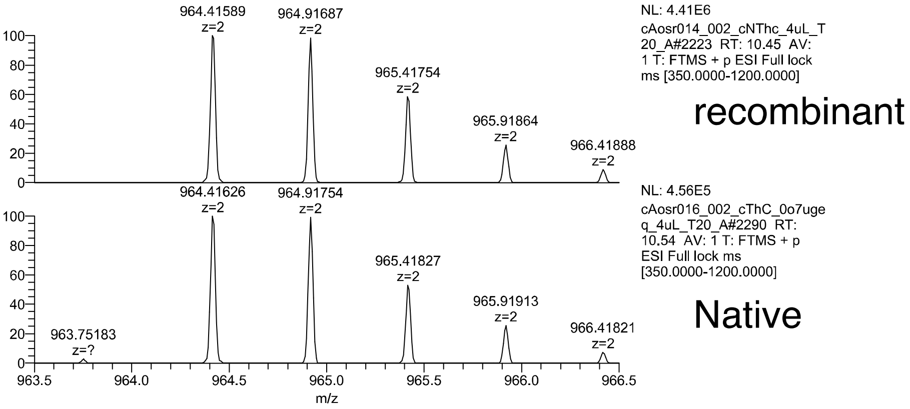

DFNDVFVLITGLVR (res 117-130)

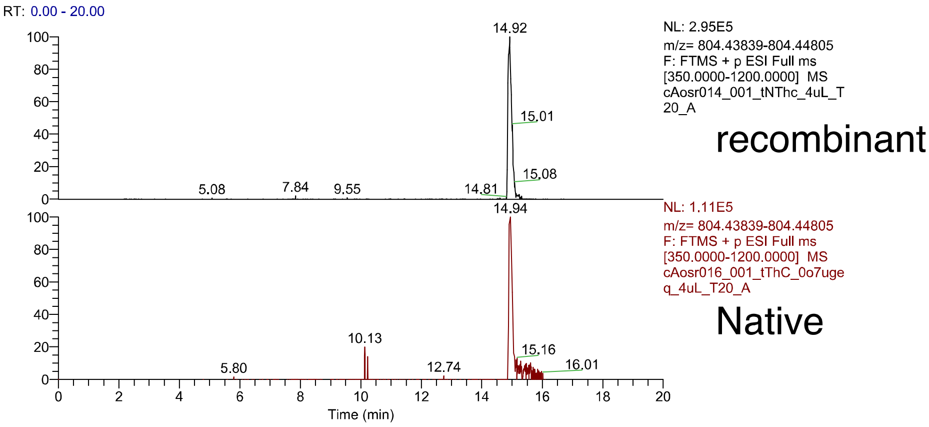

charge 2 (theoretical m/z=804.44322)
recombinant m/z=804.44360, RT=14.85, Δppm=0.42 (PD)
Native m/z=804.44287, RT=14.94, Δppm=-0.49 (PD)

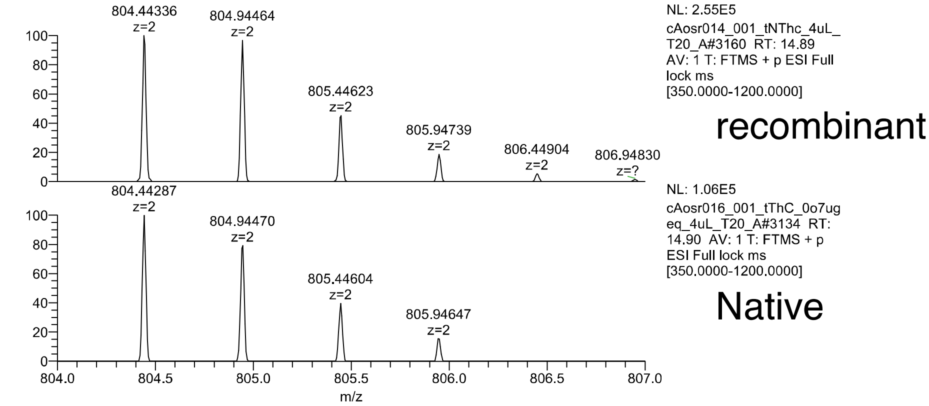

VLITGLVRG (res 123-131)

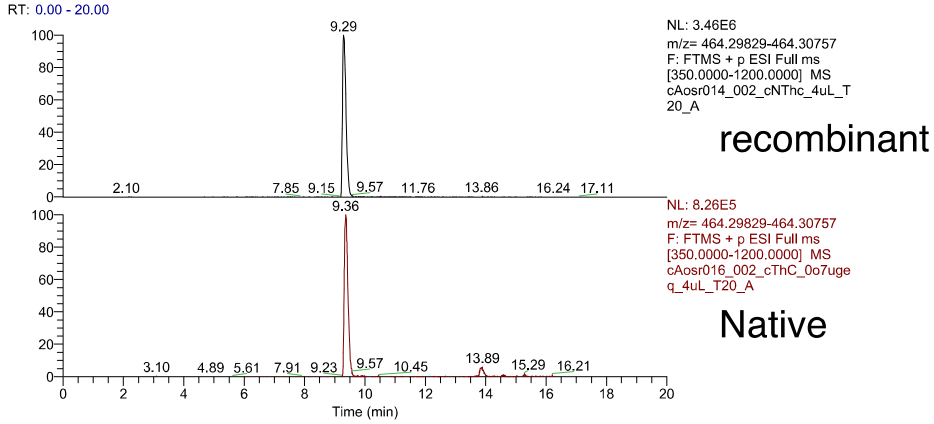

charge 2 (theoretical m/z=464.30293)
recombinant
Native m/z=464.40289, RT=9.30, Δppm=-0.14 (PD)

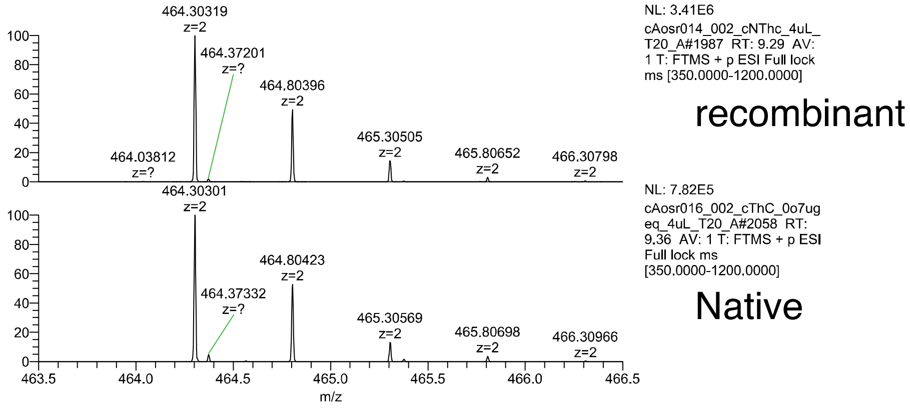

Supplementary Figure 6. Sequence determination of ThC continued

Mass spectrometry confirmation of the identified peptides of nThC compared with rThC. Extracted ion chromatograms (XICs) of the identified peptides of rThC and nThC were shown as black and red, respectively. MS spectra of the charged peptide ion from rThC (upper) and nThC (bottom) at the XIC peak are shown, respectively. RT, retention time; PD, the peptide identified by Proteome Discoverer 1.4.0.288 (Thermo Scientific); PEAKS, the peptide identified by PEAKS studio 10.0 Build 20190129 (Bioinformatics Solutions); Δppm, parts per million of the mass differences between observed and theoretical m/z values. The m/z, RT, and Δppm of the peptide identified by PD or PEAKS were indicated. Carbamidomethyl-Cys residue was shown as lower case.

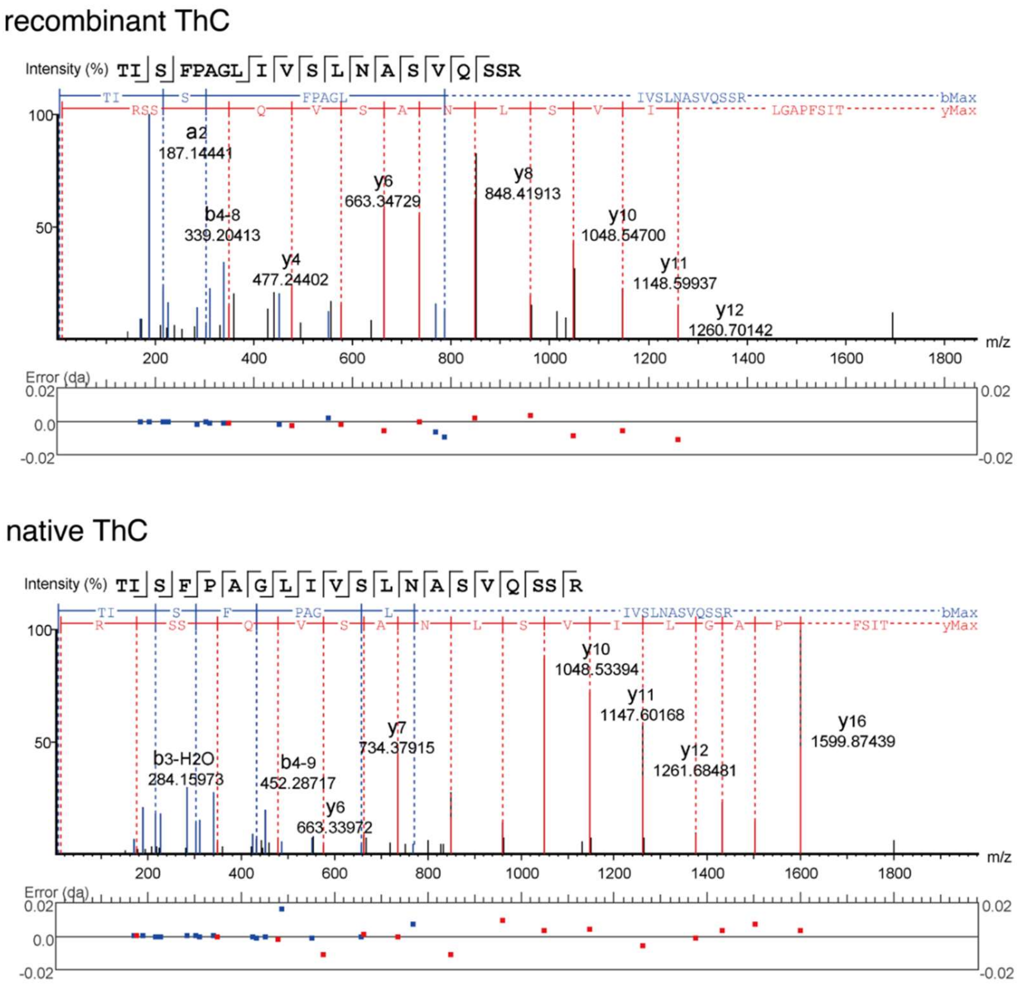

Supplementary Figure 7. Sequence determination of ThC continued

Annotated MS/MS fragmentation spectra for a putative N-terminal 9-28 residues, TISFPAGLIVSLNASVQSSR, derived from rThC (upper panel) and nThC (lower panel). MS/MS spectra were deconvoluted into singly charged ions from the observed spectra and theoretical m/z values for fragment ions were assigned for each peak. The identified matches for the N-terminal-containing ions are shown in blue and those for the C-terminal-containing ions in red. The m/z differences between theoretical and observed values for all assigned peaks were displayed in error map and were less than 0.02 Da. The MS/MS spectra confirmed that 25th residue was clearly assigned as Q. The exact masses and precursor isotope distribution of MS1 spectrum for the peptides derived from nThC and rThC (1-28 residues, 25Q) was also identical (Supplementary Figure 6), in line with above sequence analyses confirming that 25th residue the N terminal sequence is Q, not E.

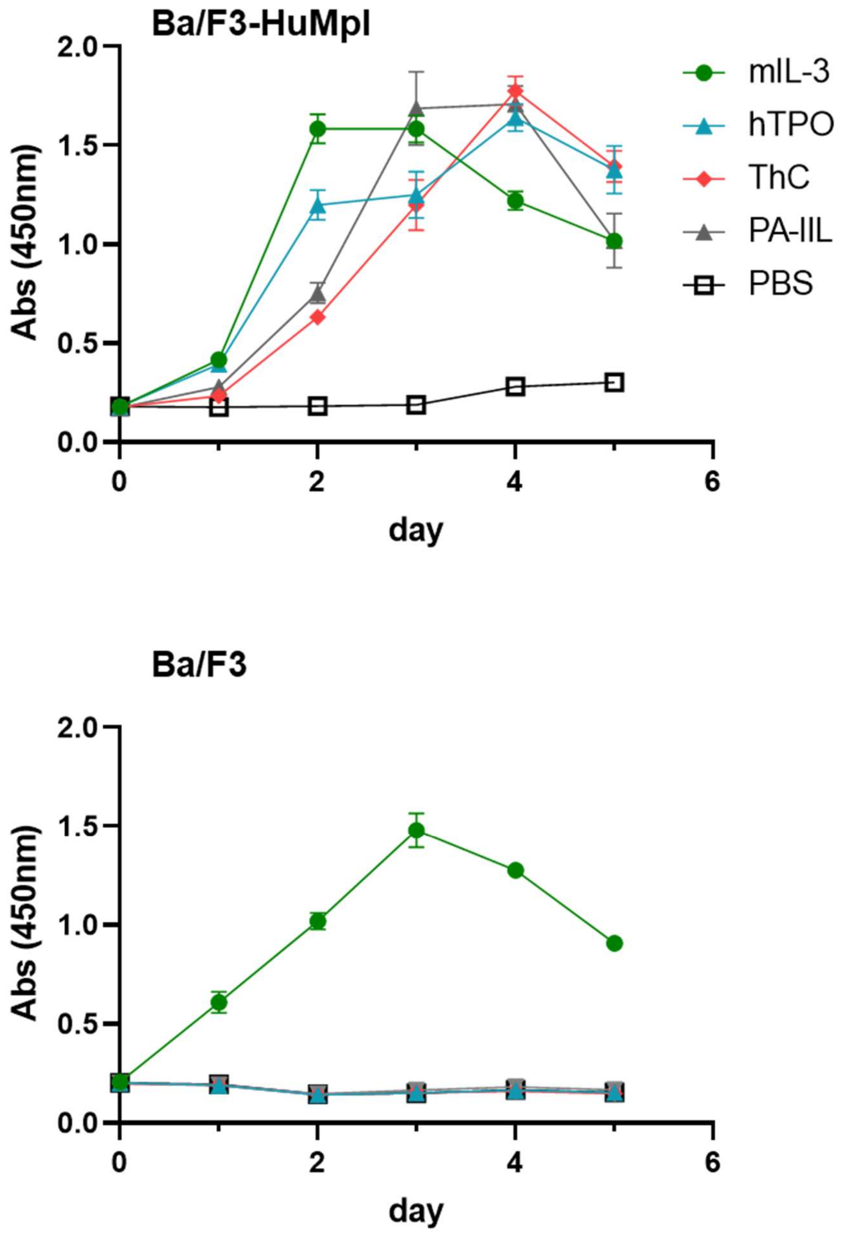

Supplementary Figure 8. Cell proliferation assay for Ba/F3 and Ba/F3-HuMpl cells in the presence or absence of ligand

Cell proliferation assay of Ba/F3 and Ba/F3-HuMpl cells in the absence or presence of the indicated ligand. murineIL-3 (10 ng/mL), humanTPO (10 ng/mL), ThC (0.5 μg/mL), PA-IIL (30 μg/mL). *n*=5 per data point. bars indicate mean ± SD.

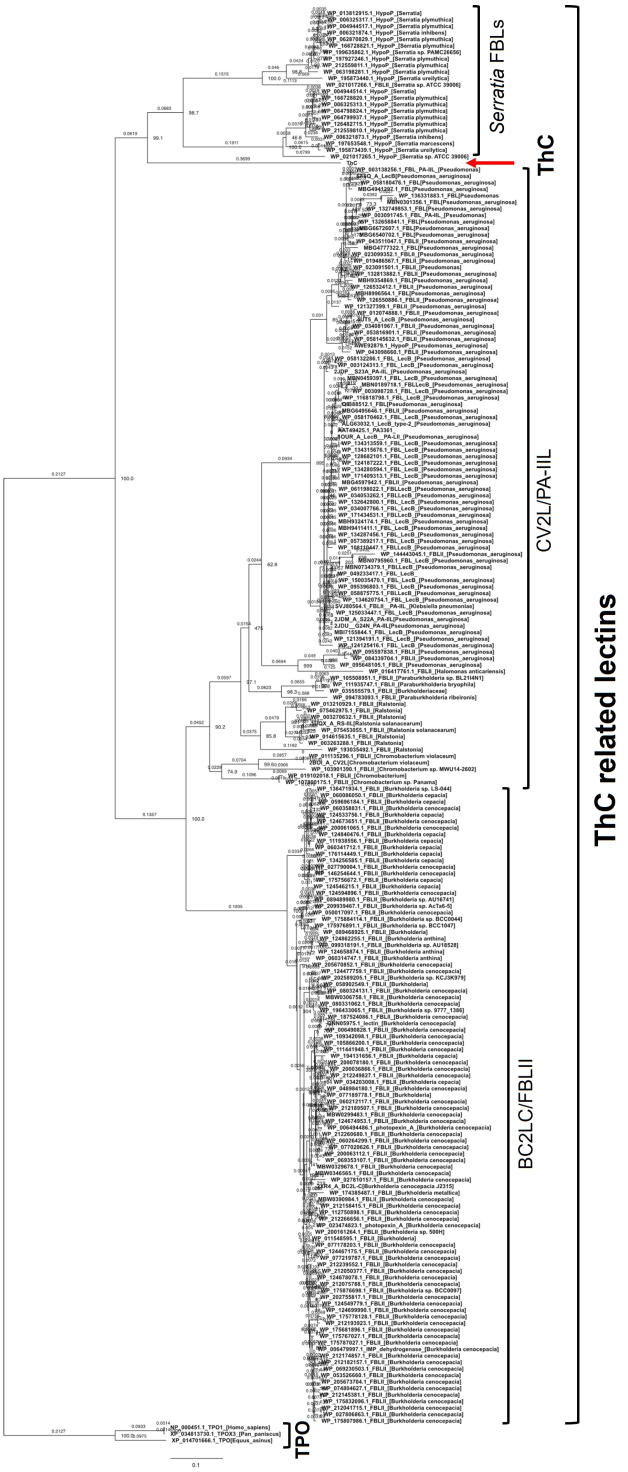

Supplementary Figure 9. Phylogenetic tree of ThC and putative bacterial fucose binding lectins and TPO

Bootstrap probabilities of 1,000 replications are shown at each node. A scale bar indicates the genetic distance represented by the horizontal branches.

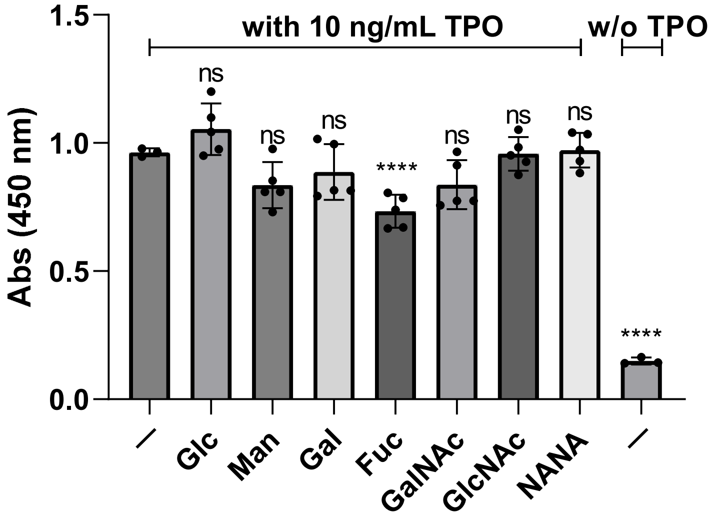

Supplementary Figure 10. Relative cell growth by TPO in the presence of various sugars in Ba/F3-HuMpl cells

Positive control contains TPO (10 ng/mL) and negative control without agonist. Each 10 mM of sugar was present. *n*=5 per data point except positive and negative controls where *n*=3. bars indicate mean ± SD. One-way ANOVA followed by a Dunnett's test for multiple group comparison, ****P < 0.001 vs. positive control, ns, not significant.

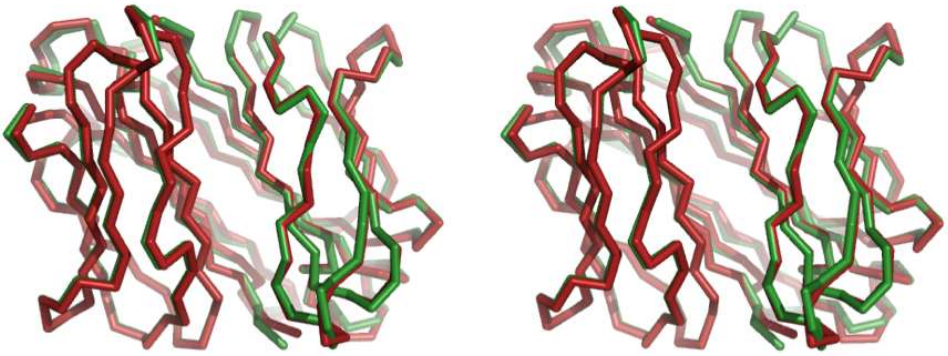

Supplementary Figure 11. Stereo representation of superposition of nThC (green) and rThC (red)

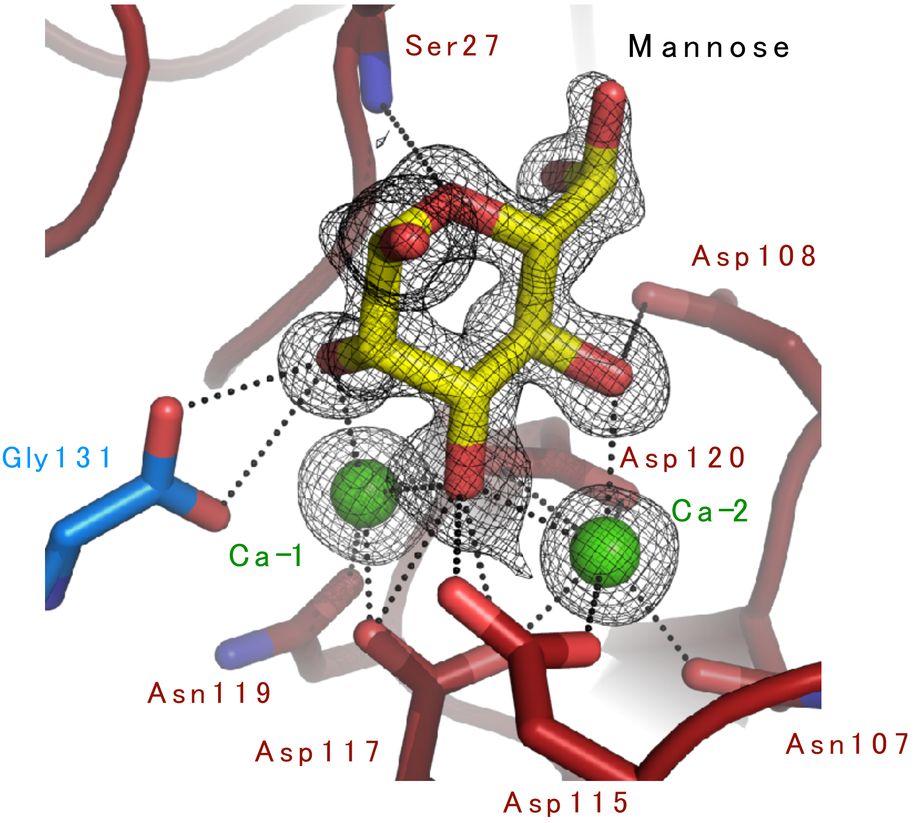

Supplementary Figure 12. Close-up view of carbohydrate binding site in complex with rThC and D-mannose
Fo-Fc map of the carbohydrates and Ca^{2+} ions contoured at 3.0 σ are shown. Individual protomers are shown red and blue, respectively.

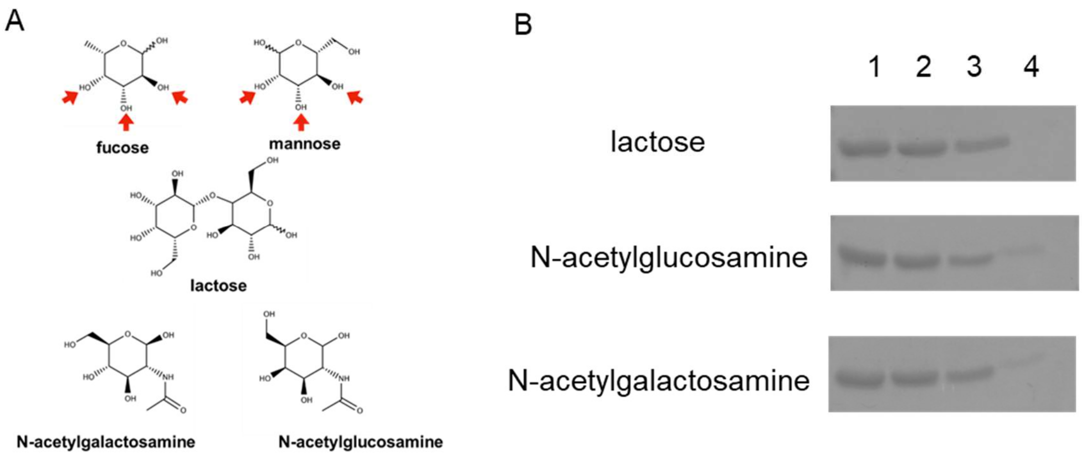

Supplementary Figure 13. Affinity of rThC against carbohydrate-immobilized agarose gel

Chemical structure of the carbohydrates tested (A) and their binding with rThC (B). (A) fucose, mannose, lactose, N-acetylgalactosamine, and N-acetylglucosamine. Red arrows indicate hydroxyl groups which are recognized by rThC and Ca^{2+} ions. (B) Binding of rThC to the lactose, N-acetylglucosamine-, and N-acetylgalactosamine-immobilizing resin in the presence of 5 mM $CaCl_2$. Lane 1: rThC standard, 2: flow through fraction, 3: wash fraction, 4: rThC bound on the resin. Experiments in B were performed in triplicates. Uncropped gel images are provided as a Source Data file.

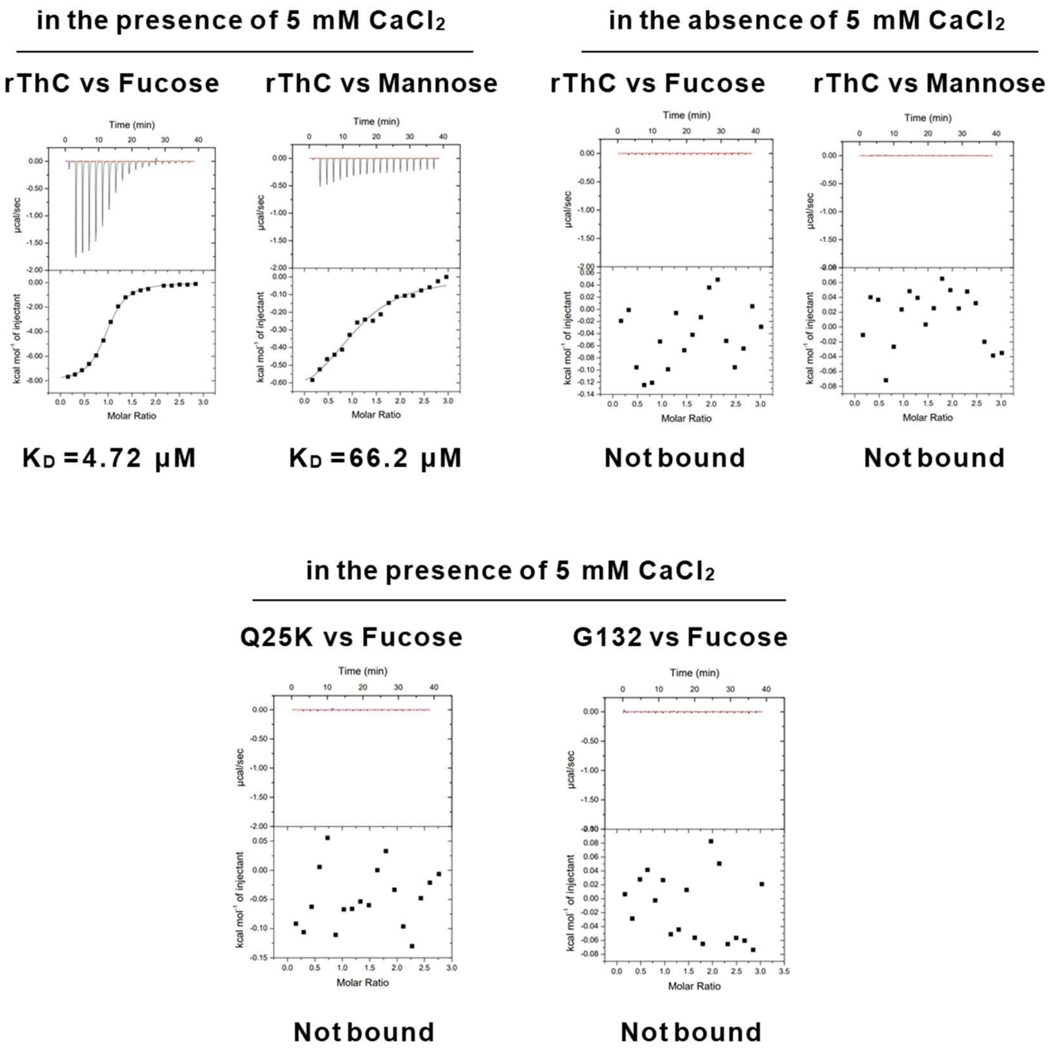

Supplementary Figure 14. Results of ITC for rThC and mutants

rThC (top) and mutants (bottom). K_D values are shown at the bottom. Thermodynamic parameters are shown in Supplementary Table S1. Experimental condition, buffer: 20 mM HEPES-NaOH (pH 8.0), 200 mM NaCl, temperature: 25 °C, injection: 18 times of 2 µL with 120 s interval.

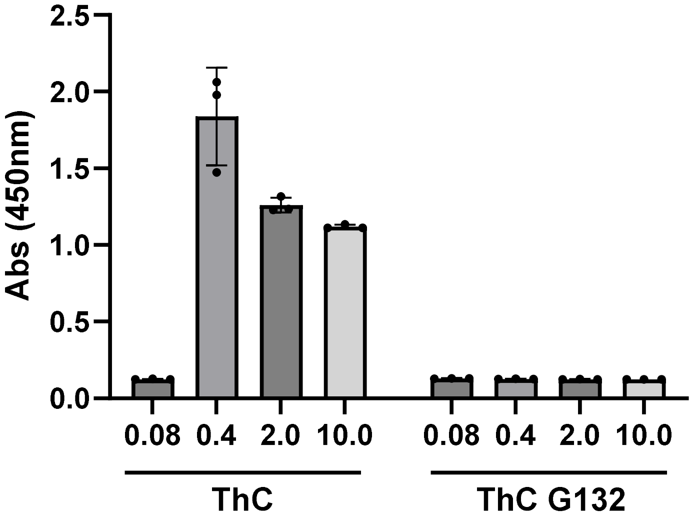

Supplementary Figure 15. Effect of rThC G132 on cell proliferation of Ba/F3-HuMpl cells

Cells were cultured with indicate concentration (μg/mL) ThC for 4 days. *n*=3 per data point. bars indicate mean ± SD.

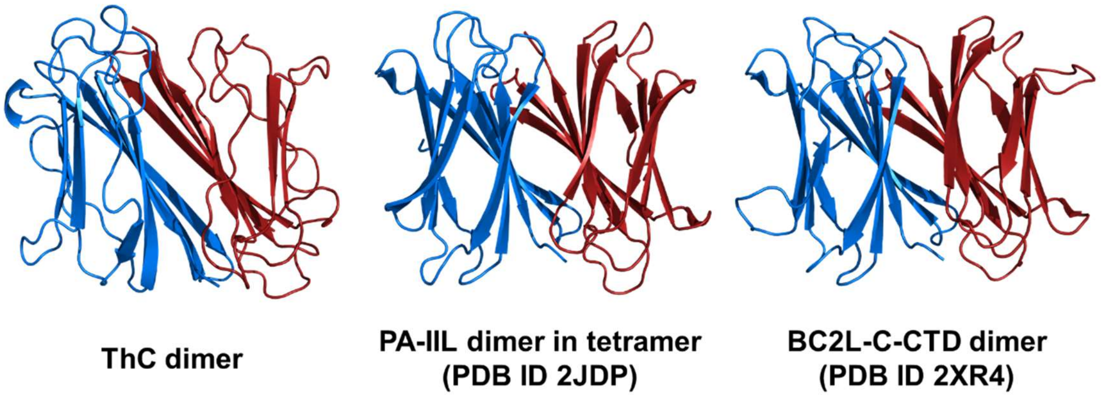

Supplementary Figure 16. Structural comparison between ThC dimer, PA-IIL dimer (PDB ID 2JDP), and BC2L-C-CTD dimer (PDB ID 2XR4)

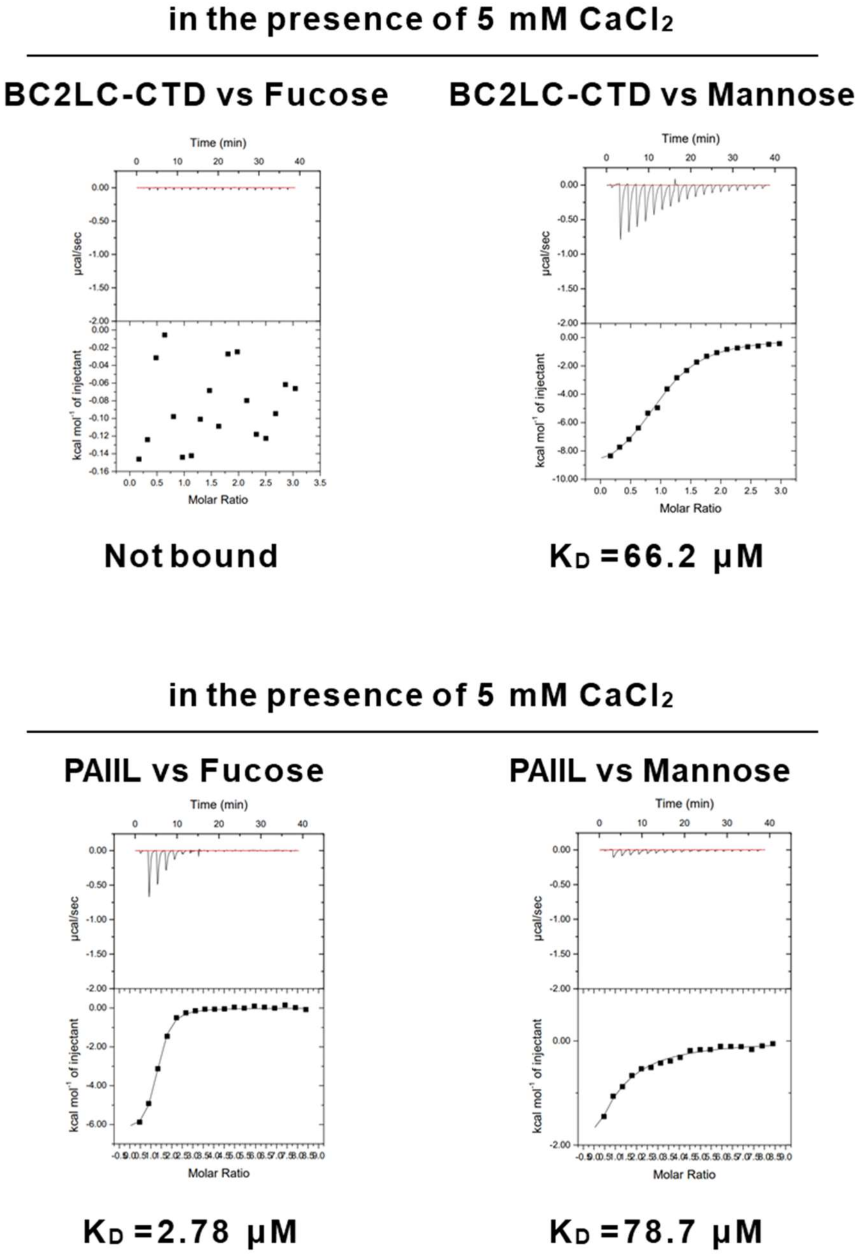

Supplementary Figure 17. Results of ITC of BC2LC-CTD (top) and PA-IIL (bottom)

K_D values are shown at the bottom. Thermodynamic parameters are shown in Supplementary Table 1. Experimental condition, buffer: 20 mM HEPES-NaOH (pH 8.0), 200 mM NaCl, temperature: 25 °C, injection: 18 times of 2 µL with 120 s interval.

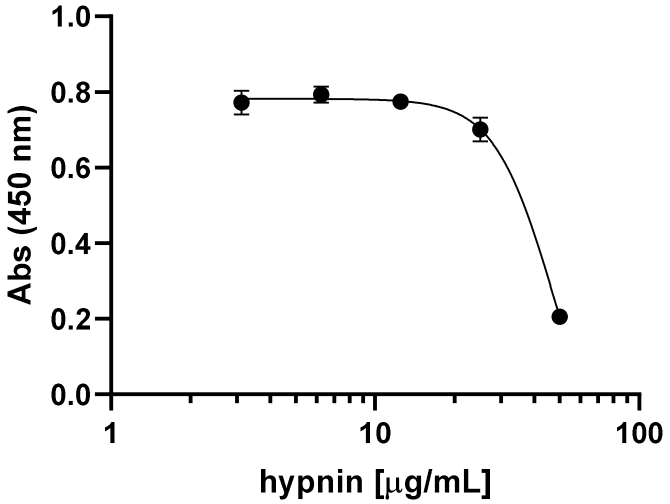

Supplementary Figure 18. Effect of hypnin on Ba/F3 cell proliferation

Cells were cultured with 10 ng/mL mIL-3 for 4 days. IC_{50} > 47 μg/mL. *n*=5 per data point. bars indicate mean ± SD.

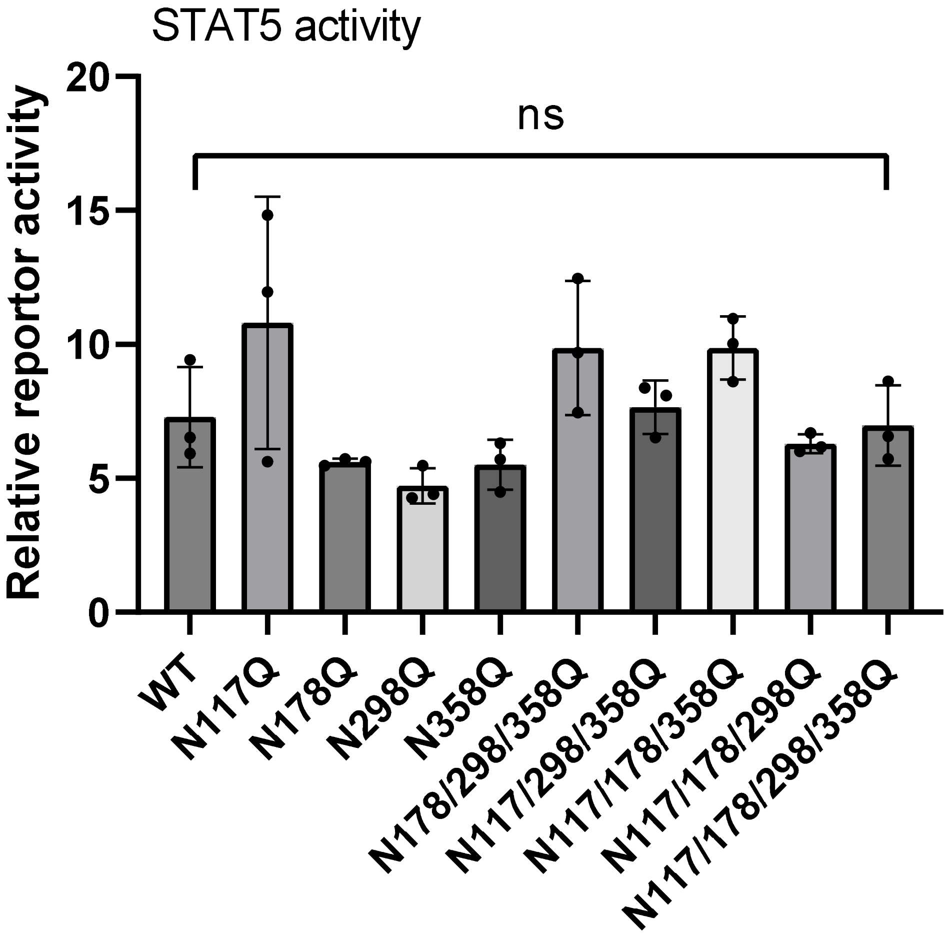

Supplementary Figure 19. Comparable STAT5 activation by TPO with MPL bearing N/Q mutation

STAT5 reporter activity in HEK293T cells expressing a series of N/Q mutant constructs when stimulated by TPO (10 ng/mL). ns = no significance. *n*=3 per data point. bars indicate mean ± SD. One-way ANOVA followed by a Dunnett's test for multiple group comparison, ns: not significant

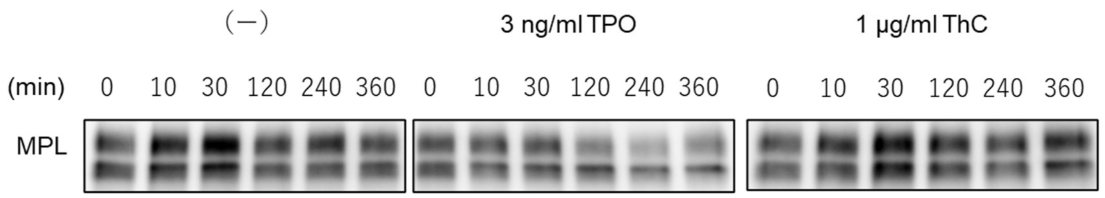

Supplementary Figure 20. Western Blot data for Fig 5B.

0, 10, 30, 120, 240 and 360 min after the addition of ThC (1 μg/mL), TPO (3 ng/mL), control (-, No agonist). Levels of cell surface MPL was determined and depicted. Three independent experiments were carried out. Source data are provided as a Source Data file.

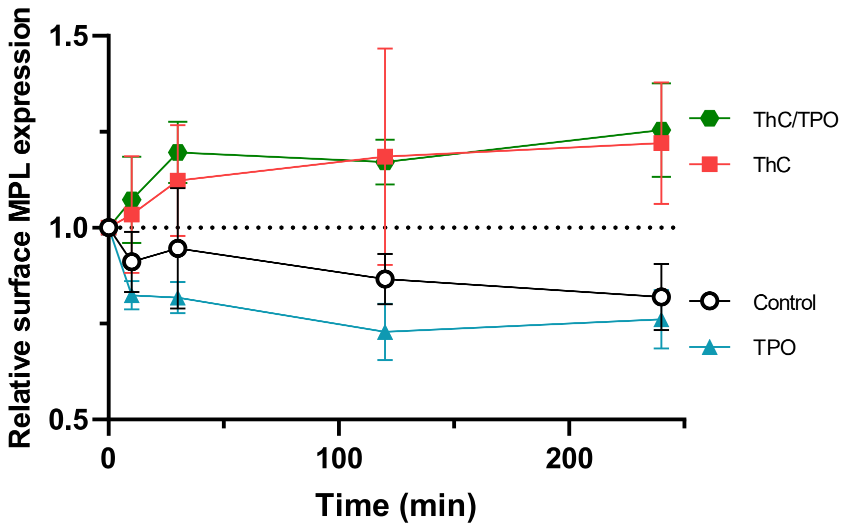

Supplementary Figure 21. Interference of the TPO-dependent internalization of MPL from cell surface by ThC

0, 10, 30, 120 and 240min after the addition of ThC (0.1 μg/mL), ThC/TPO (0.1 μg/mL and 0.2 ng/mL, respectively), TPO (0.2 ng/mL) and control (No agonist), levels of cell surface MPL was determined and depicted. *N*=3 per data point. Bars indicate mean ± SD.

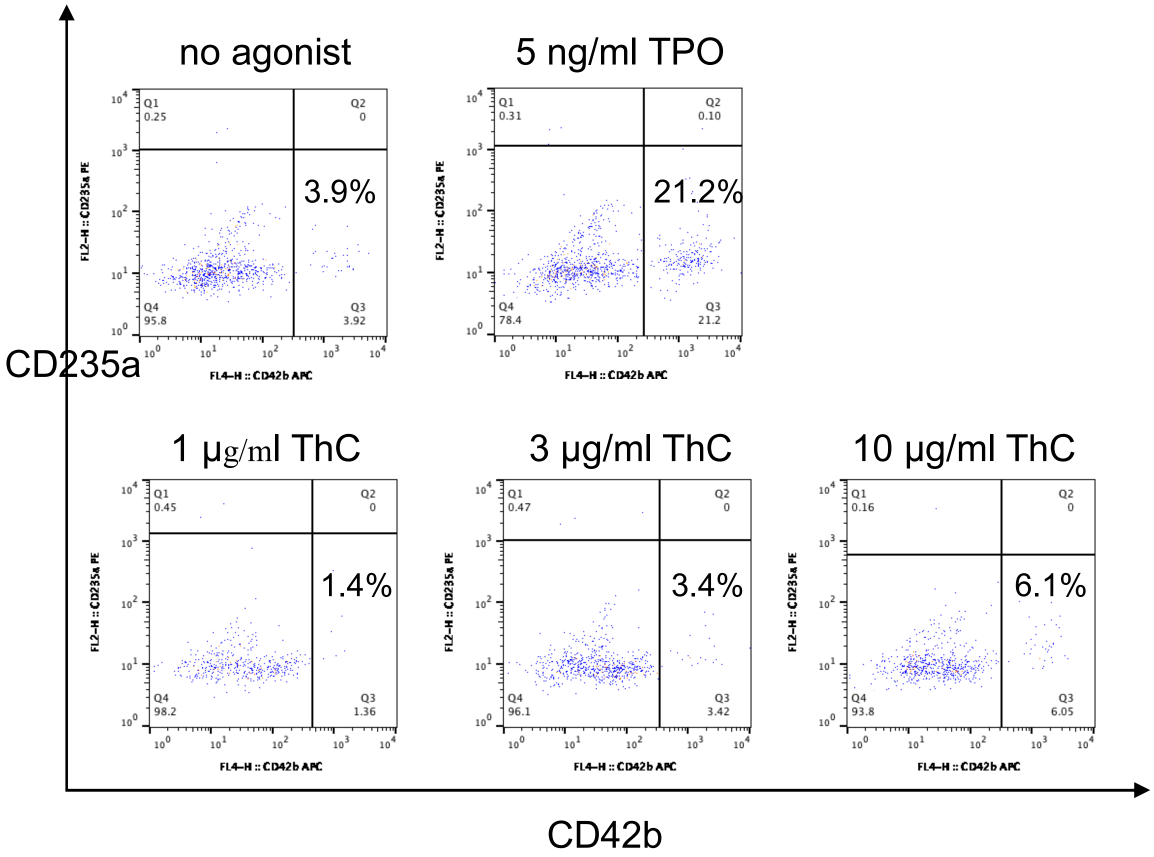

Supplementary Figure 22. Limited effect of ThC on the induction of megakaryocytic cells in iPS-derived hematopoietic stem/progenitor cells

Hematopoietic stem/progenitor cells (HPSCs) induced from iPS cells [4] were cultured in Iscove-modified Dulbecco media (Thermo Fisher #12440-053) supplemented with 15% FBS (Biowest #S1530), 1 x penicillin-streptomycin-glutamine (Thermo Fisher #10378-016), 1 x Insulin-Transferrin-Selenium (Thermo Fisher #41400-045), 0.45 mM 1-thioglycerol (Sigma #M6145), and 50 μg/mL ascorbic acid (Sigma #A4544) in the presence or absence of indicated agonist. 7 days after the differentiation, cells were collected, stained with anti-human CD42b-APC (BD Bioscience #551061) and anti-human CD235a-PE (BD Bioscience #555570), and analyzed by FACSCalibur (BD Biosciences). Flow cytometric plots showed limited effect of ThC on the induction of megakaryocytic cells defined by the expression of CD42b. While thrombopoietin (TPO), the natural ligand for MPL, induced megakaryocytic differentiation. Experiments were performed three times and obtained reproducible results.

Supplementary References

1 Sherman, W., Day, T., Jacobson, M. P., Friesner, R. A. & Farid, R. Novel procedure for modeling ligand/receptor induced fit effects. *Journal of medicinal chemistry* **49**, 534-553 (2006).

2 Pokorná, M. *et al.* Unusual entropy-driven affinity of Chromobacterium violaceum lectin CV-IIL toward fucose and mannose. *Biochemistry* **45**, 7501-7510 (2006).

3 Sabin, C. *et al.* Binding of different monosaccharides by lectin PA-IIL from Pseudomonas aeruginosa: thermodynamics data correlated with X-ray structures. *FEBS Lett.* **580**, 982-987 (2006).

4 Takei, H. *et al.* Skewed megakaryopoiesis in human induced pluripotent stem cell-derived haematopoietic progenitor cells harbouring calreticulin mutations. *Br. J. Haematol.* **181**, 791-802 (2018).
