# Supplementary material for: A marine sponge-derived lectin reveals hidden pathway for thrombopoietin receptor activation
Source: Nat Commun. 2022 Nov 25;13:7262. doi: 10.1038/s41467-022-34921-2 (PMC9700728; doi:10.1038/s41467-022-34921-2)
Supplement: Supplementary file 3 — Reporting Summary [file 41467_2022_34921_MOESM3_ESM.pdf]

nature portfolio

Corresponding author(s): Ryuichi Sakai
Marito Araki
Yoshikazu Tanaka

Last updated by author(s): Mar 30, 2022

Reporting Summary

Nature Portfolio wishes to improve the reproducibility of the work that we publish. This form provides structure for consistency and transparency in reporting. For further information on Nature Portfolio policies, see our Editorial Policies and the Editorial Policy Checklist.

Statistics

For all statistical analyses, confirm that the following items are present in the figure legend, table legend, main text, or Methods section.

n/a	Confirmed	
☐	☒	The exact sample size (n) for each experimental group/condition, given as a discrete number and unit of measurement
☐	☒	A statement on whether measurements were taken from distinct samples or whether the same sample was measured repeatedly
☐	☒	The statistical test(s) used AND whether they are one- or two-sided<br>*Only common tests should be described solely by name; describe more complex techniques in the Methods section.*
☒	☐	A description of all covariates tested
☒	☐	A description of any assumptions or corrections, such as tests of normality and adjustment for multiple comparisons
☐	☒	A full description of the statistical parameters including central tendency (e.g. means) or other basic estimates (e.g. regression coefficient) AND variation (e.g. standard deviation) or associated estimates of uncertainty (e.g. confidence intervals)
☐	☒	For null hypothesis testing, the test statistic (e.g. F, t, r) with confidence intervals, effect sizes, degrees of freedom and P value noted<br>*Give P values as exact values whenever suitable.*
☒	☐	For Bayesian analysis, information on the choice of priors and Markov chain Monte Carlo settings
☒	☐	For hierarchical and complex designs, identification of the appropriate level for tests and full reporting of outcomes
☒	☐	Estimates of effect sizes (e.g. Cohen's d, Pearson's r), indicating how they were calculated

Our web collection on statistics for biologists contains articles on many of the points above.

Software and code

Policy information about availability of computer code

Data collection	Thermo Xcalibur 3.0.63 (LC-MS)
Data analysis	The following softwares were used in this study: GraphPad Prism 8.0.3, Origin 7 SR4 v7.0552(B552)<br>HKL2000, XDS (Version March 15, 2019), HKL2MAP, phenix.mr, phenix.autosol, phenix.refine in the PHENIX program suite (version 1.14-3260), phniex.map in the PHENIX program suite (version 1.19.2-4158), PyMOL (version 2.2.0), Proteome Discoverer 1.4.0.288, PEAKS Studio 10.0 build 20190129.

For manuscripts utilizing custom algorithms or software that are central to the research but not yet described in published literature, software must be made available to editors and reviewers. We strongly encourage code deposition in a community repository (e.g. GitHub). See the Nature Portfolio guidelines for submitting code & software for further information.

Data

Policy information about availability of data

All manuscripts must include a data availability statement. This statement should provide the following information, where applicable:

- Accession codes, unique identifiers, or web links for publicly available datasets
- A description of any restrictions on data availability
- For clinical datasets or third party data, please ensure that the statement adheres to our policy

Coordinates and structure factors have been deposited in the Protein Data Bank under accession codes 7F9F[https://www.rcsb.org/structure/7F9F] (nThC), 7F91 [https://www.rcsb.org/structure/7F91] (Met substituted rThC), 7F9G[https://www.rcsb.org/structure/7F9G] (rThC in complex with Ca2+ and fucose), 7FBL[https://www.rcsb.org/structure/7FBL] (rThC in complex with Ca2+ and mannose), and 7F9J[https://www.rcsb.org/structure/7F9J] (rThC Q25K in complex with Ca2+). All

other data are available in the main text or the supplementary materials. SwissProt accession number: C0HM62 for Thrombocorticin was assigned. Source data are provided with this paper.

Field-specific reporting

Please select the one below that is the best fit for your research. If you are not sure, read the appropriate sections before making your selection.

☒ Life sciences ☐ Behavioural & social sciences ☐ Ecological, evolutionary & environmental sciences

For a reference copy of the document with all sections, see nature.com/documents/nr-reporting-summary-flat.pdf

Life sciences study design

All studies must disclose on these points even when the disclosure is negative.

Sample size	More than three experiments are performed to gain statistical data. This sample size was used in our previous experiments, for example Blood 127, 1307-1316 (2016).
Data exclusions	No data were excluded
Replication	All measurements are replicated with at least three sets of experiments. P values, 95% confidence intervals, or table of analysis are given either in main text or supporting information.
Randomization	Cells were randomly allocated from the culture flask to multi well plates to perform cell proliferation experiments.
Blinding	Not relevant to this study. All data collected are either controlled (cell proliferation assays), or quantitative (X-ray diffraction, LC-MS), and no subjective judgments are required. All the data used in this paper are provided in the paper, supporting information, source data and public databases.

Reporting for specific materials, systems and methods

We require information from authors about some types of materials, experimental systems and methods used in many studies. Here, indicate whether each material, system or method listed is relevant to your study. If you are not sure if a list item applies to your research, read the appropriate section before selecting a response.

Materials & experimental systems

n/a	Involved in the study	
☐	☒	Antibodies
☐	☒	Eukaryotic cell lines
☒	☐	Palaeontology and archaeology
☐	☒	Animals and other organisms
☒	☐	Human research participants
☒	☐	Clinical data
☒	☐	Dual use research of concern

Methods

n/a	Involved in the study	
☒	☐	ChIP-seq
☒	☐	Flow cytometry
☒	☐	MRI-based neuroimaging

Antibodies

Antibodies used

phospho-JAK2 Cell Signaling Technology #3771 Dilution 1:1000
phospho-STAT5 Cell Signaling Technology #9359 Dilution 1:1000
phospho-ERK1/2 Cell Signaling Technology #9101 Dilution 1:1000
phospho-AKT Cell Signaling Technology #4060 Dilution 1:1000
JAK2 Cell Signaling Technology #3230 Dilution 1:2000
STAT5 Cell Signaling Technology #94205 Dilution 1:2000
ERK1/2 Cell Signaling Technology #9102 Dilution 1:2000
AKT Cell Signaling Technology #9271 Dilution 1:2000
MPL Merck Millipore #06-044 Dilution 1:2000
b-actin Cell Signaling Technology #4967 Dilution 1:20000

Validation

Validated by manufacturer. Details are provided in source data

Eukaryotic cell lines

Policy information about cell lines

Cell line source(s)	Ba/F3 cells , gift from Nissan Chemical Co. Ltd and Dr. Megumi Funakoshi-Tago at Faculty of Pharmacy, Keio University. iPS cells were from our previous study (Br J Haematol. 2018 Jun;181(6):791-802.)
Authentication	Not authentificated
Mycoplasma contamination	cell line was not tested for Mycoplasma contamination
Commonly misidentified lines (See ICLAC register)	not relevant to this study

Animals and other organisms

Policy information about studies involving animals; ARRIVE guidelines recommended for reporting animal research

Laboratory animals	No laboratory animals were used.
Wild animals	The marine sponge Corticium sp. was collected using SCUBA. The specimen was frozen immediately after collection and identified as indicated in our previous publication in ref 12. All sponge material was used for isolation of the lectin.
Field-collected samples	The sponge specimen was kept frozen at -20 degree until use.
Ethics oversight	The sponge specimen was collected under permission of Chuuk State Department of Marine Resources.

Note that full information on the approval of the study protocol must also be provided in the manuscript.
